# Absence of *AKT1* Mutations in Glioblastoma

**DOI:** 10.1371/journal.pone.0005638

**Published:** 2009-05-20

**Authors:** Fonnet E. Bleeker, Simona Lamba, Carlo Zanon, Angela A. van Tilborg, Sieger Leenstra, Dirk Troost, Theo Hulsebos, W. Peter Vandertop, Alberto Bardelli

**Affiliations:** 1 Neurosurgical Center Amsterdam, Academic Medical Center, University of Amsterdam, Amsterdam, The Netherlands; 2 Laboratory of Molecular Genetics, The Oncogenomics Center, Institute for Cancer Research and Treatment, University of Torino Medical School, University of Torino, Medical School, Candiolo (TO), Italy; 3 Department of Pathology, Erasmus Medical Center, Rotterdam, The Netherlands; 4 Department of Neurosurgery, Erasmus Medical Center, Rotterdam, The Netherlands; 5 Department of Neurosurgery, St. Elisabeth Ziekenhuis Tilburg, Tilburg, The Netherlands; 6 Department of Neuropathology, Academic Medical Center, University of Amsterdam, Amsterdam, The Netherlands; 7 Department of Neurogenetics, Academic Medical Center, University of Amsterdam, Amsterdam, The Netherlands; 8 Neurosurgical Center Amsterdam, Location VU University Medical Center, Amsterdam, The Netherlands; 9 FIRC Institute of Molecular Oncology, Milan, Italy; Deutsches Krebsforschungszentrum, Germany

## Abstract

**Background:**

Oncogenic activation of the PI3K signalling pathway plays a pivotal role in the development of glioblastoma multiforme (GBM). A central node in PI3K downstream signalling is controlled by the serine-threonine kinase AKT1. A somatic mutation affecting residue E17 of the *AKT1* gene has recently been identified in breast and colon cancer. The E17K change results in constitutive AKT1 activation, induces leukaemia in mice, and accordingly, may be therapeutically exploited to target the PI3K pathway. Assessing whether AKT1 is activated by somatic mutations in GBM is relevant to establish its role in this aggressive disease.

**Methodology/Principal Findings:**

We performed a systematic mutational analysis of the complete coding sequence of the AKT1 gene in a panel of 109 tumor GBM samples and nine high grade astrocytoma cell lines. However, no somatic mutations were detected in the coding region of *AKT1*.

**Conclusions/Significance:**

Our data indicate that in GBM oncogenic deregulation of the PI3K pathway does not involve somatic mutations in the coding region of *AKT1*.

## Introduction

A number of genetic and functional evidences have unequivocally established the importance of the PI3K pathway in human cancer [1], [2]. For example oncogenic deregulation of the PI3K pathway plays a central role in the development of Glioblastoma Multiforme (GBM) as shown by the fact that many of its members are genetically altered [3], [4]. Two main regulators, the lipid kinase PIK3CA and the lipid phosphatase PTEN, control this signalling pathway. We and other have shown that the *PIK3CA* gene is mutated in many tumour types, including GBM [4]–[7]. The corresponding mutations result in activation of the PI3K catalytic activity and constitutive downstream signalling. The tumour suppressor gene PTEN encodes for a lipid phopshatase which counteracts the effect of PI3K thus negatively controlling signalling. *PTEN* is mutationally and transcriptionally inactivated in many different tumour types, including GBM [8]. In most tumour lineages, including GBMs *PIK3CA* and *PTEN* mutations occur in a mutually exclusive manner [6], [9]. This suggests that they exert overlapping cellular functions, and in fact, both control the cellular levels of phosphatidylinositol-3-phosphate (PIP3) [10], [11]. Other mechanisms of activation of the PI3K pathway include alterations in tyrosine kinase receptors acting upstream in the signalling cascade. This is the case for the receptor tyrosine kinase *EGFR* which can be activated by gene amplification and/or mutations [12]. Both missense point mutations and large extracellular domain deletions (*EGFRvIII*, which due to alternative splicing misses exon 2–7) affecting the *EGFR* gene have been reported at considerable frequency in GBM and result in constitutive activation of the receptor and of the underlying PI3K pathway [12].

Downstream in the signaling cascade, PI3K and PTEN control PIP3, which activates downstream effector molecules, such as the serine-threonine kinase AKT. A recent sequencing study led to the identification of oncogenic somatic mutations in the pleckstrin homology domain of *AKT1* in breast, colon and ovarian cancer [13]. Interestingly, in all cases examined the same mutation E17K was identified. This mutation alters the electrostatic interactions of the pocket and constitutively activates AKT1 in a PI3K-independent manner. By this mechanism, it transforms rodent cells in vitro and can induce leukaemia in mice [13]. Recently, we and others assessed the mutational status of the E17K mutation in different tumor types [14]–[17], confirming the mutations in breast and colon, and most interestingly revealing mutations in lung cancer [14], [15]. On the contrary, we did not detect any mutations affecting the hotspot residue E17 in a panel of 128 GBMs [15]. We noted that, GBMs do exhibit a different mutation spectrum for some genes compared to other tumor types. For example, most *EGFR* and *ERBB2* mutations in lung cancer are found in the kinase domain [18], [19], whereas these genes are predominantly mutated in the extracellular domain in GBM [12], [20]. This led us to speculate that GBMs might bear mutations in other regions of the *AKT1* gene. To definitively assess this hypothesis, we successfully sequenced the complete coding sequence of *AKT1* in 109 GBM tumor samples and nine high grade astrocytoma cell lines.

## Results and Discussion

We sequenced the complete coding sequence of the *AKT1* gene in a set of 109 GBM tumors and 9 high grade astrocytoma cell lines. Primers were designed to amplify and sequence the genomic region corresponding to all coding exons of *AKT1*, including exon 4, where the E17K residue is located. Amplicons included at least 15 intronic bases at both the 5′ and 3′ ends encompassing the splicing donor and acceptor sites. A total of 1535 PCR products, spanning 628 kb of tumour genomic DNA, were generated and subjected to direct sequencing. Sequencing was performed single stranded with either forward or reverser primer. Identified changes were independently confirmed by another round of PCR and sequencing.

Previous work focusing on the mutational analysis of exon 4 did not identify any E17K mutation in 128 GBM samples [15]. Importantly, we extended the mutational analysis for these tumors to all other coding exons of *AKT1*, but no somatic mutations were found.

Our tumor set was validated by previous mutational profiling of common cancer genes, including the *IDH1* gene [21]. Furthermore, we found a number of previous reported SNPs in our samples (rs17846822, rs34664585, rs3730358, rs3730368, rs2494737, rs2494735, rs34670300, rs3730361, rs3730329, rs3730329, rs2494732). In addition, we identified three novel germline changes (IVS8-12C>T, R200H and W333*) at low frequency, they were found only once in different samples. Our work is focused on somatic mutations and therefore, we did not study these changes in further detail.

While previous studies have focused mainly on the hotspot mutation site *AKT1^E17^*or the serine and threonine phosphorylation sites [3], [15], [17], this work is the first to show in a large panel of GBMs that the coding sequence of the AKT1 is not somatically mutated in this tumour lineage. Although the promoter region and/or the 5′ and 3′ UTR of *AKT1* may contain mutations, our data indicate that in GBM oncogenic deregulation of the PI3K pathway does not involve mutations in the coding region of *AKT1*.

## Materials and Methods

### Tumor sample, Ethics Statement and Isolation of Genomic DNA

All samples were collected from patients undergoing brain tumor surgery in the Academic Medical Center (Amsterdam, The Netherlands). Oral consent for removal of the tissue and its storage in the tumor bank for research purposes was obtained and documented in the patient's medical chart. Individual consent for this specific project was waivered by our ethics committee because the research was performed on ‘waste’ material, stored in a coded fashion. Patient characteristics are displayed in Table 1. Tumor samples were included only if at least 80% of the sample consisted of cancer cells, as verified by H&E staining.

**Table 1:** Characteristics of 109 GBM tumor samples.

GBM subclasses	primary	94
	secondary	15
Patient sex	female	47
	male	62
Patient age	mean age (years)	53.6
	median age (years)	55

Nine astrocytoma cell lines were included: the cell lines U87MG, U118MG, U251MG, U373MG, T98G (ATCC, Middlesex, United Kingdom), SKMG-3 (a gift of Dr Christopher Y. Thomas, University of Virginia Division of Hematology/Oncology, Charlottesville, VA), SF763 (gift of Dr M.L. Lamfers, Department of Neurosurgery, Free University, Amsterdam, The Netherlands), SF126 (a gift of Dr C. Van Bree, University of Amsterdam, Laboratory for Experimental Oncology and Radiation Biology, Amsterdam, The Netherlands), Gli-6 was derived from our own laboratory. Genomic DNA was isolated as previously described [22].

### PCR, Sequencing and Analysis

PCR primers were designed using Primer 3 (http://frodo.wi.mit.edu/cgi-bin/primer3/primer3_www.cgi), and synthesized by Invitrogen/Life Technologies, Inc. (Paisley, England) (Table 2). PCR primers that amplify the selected exons and the flanking intronic sequences, including splicing donor and acceptor regions, were used and PCR products were on average 381 bps in length. PCRs were performed in both 384- and 96-well formats in 5- or 10-uL reaction volumes, respectively, containing 0.25 mmol/L deoxynucleotide triphosphates, 1 umol/L each of the forward and reverse primers, 6% DMSO, 1× PCR buffer, 1 ng/uL DNA, and 0.05 unit/uL Platinum Taq (Invitrogen/Life Technologies). A touchdown PCR program was used for PCR amplification (Peltier Thermocycler, PTC-200, MJ Research, Bio-Rad Laboratories, Inc., Italy).

**Table 2:** Primer details to sequence the coding sequence of AKT1.

Exon	Forward Primer Sequence	Reverse Primer Sequence	Sequencing Primer Sequence
3	GTCAGAGAGCTTAGAGGGATGG	GGCACAGGCACTCACAGA	AGTGGGTCTCTGGCTCACC
4	AGTCTGCCTTCCCGTTGAC	CAGCCAGTGCTTGTTGCTT	GTTTCTGTCGCTGGCCCTA
5	AGGCTTGGAGAGAGGAAGAGAT	GGAGTGAGGATGGCTACAGG	CTGGTGGGTGGTATGCAAG
6	AGGGCTGTCTCTGGGAACC	TGGAGTGCTGAGTGTCTCCTG	GGGTGGGTGAAAGACGTG
7	GTTCCCTGTAAGCCTGGACTC	TAAAGCAGGGCTGGGTGA	GCTCTGCCTCCGACTCTG
8	CCGCTACTACGCCATGAAGA	ACATCGTCCCCTAGAGACAGC	CATGAAGATCCTCAAGAAGGAAG
9	AACACTCCTTGGCACCTCAG	TAACTCAGCAGGAACAAGTCACC	ATCAGGCGACGTGGTATCAA
10	AGTTAGGGCTTCTGAGACTTTCC	CTTGTCCAGCATGAGGTTCTC	GTCCCTTCCCTGTGCAATG
11	CTACCTGCACTCGGAGAAGAAC	CAGGACTCGGCATCAAGG	GCACAGAGAGGACACAGCATT
12	CTGAGCACACGCAATGCT	GACATCAAGCTTTGGCTATCAGT	AATGCTGTGTCCTCTCTGTGC
13	GTGAGCTCTGTGGTGCTTTG	GCGTGAGTGTGGATATGTGG	GCCCTACATCACAGGAGGAA
14	GCTTGCTGCTCTCTGACATC	AGGCCTCTCTGAGTGTGGA	ATCCAGGTGCTTTGAAGGTCT
15	AGGTCCCTGTGTCAATCTGTG	CTCAAATGCACCCGAGAAATA	GAGGTTGGCTTCCTACTGGAG

Primers in 5′ to 3′ direction.

PCR conditions were as follows: 94°C for 2 min; three cycles of 94°C for 15 s, 64°C for 30 s, 70°C for 30 s; three cycles of 94°C for 15 s, 61°C for 30 s, 70°C for 30 s; three cycles of 94°C for 15 s, 58°C for 30 s, 70°C for 30 s; and 35 cycles of 94°C for 15 s, 57°C for 30 s, and 70°C for 30 s, followed by 70°C for 5 min and 12°C thereafter. PCR products were purified using AMPure (Agencourt Bioscience Corp., Beckman Coulter S.p.A, Milan, Italy). Cycle sequencing was carried out using BigDye Terminator v3.1 Cycle Sequencing kit (Applied Biosystems, Foster City, CA) with an initial denaturation at 97°C for 3 min, followed by 28 cycles of 97°C for 10 s, 50°C for 20 s, and 60°C for 2 min. Sequencing products were purified using CleanSeq (Agencourt Bioscience, Beckman Coulter) and analyzed on a 3730 DNA Analyzer, ABI capillary electrophoresis system (Applied Biosystems). Sequence traces were analyzed using the Mutation Surveyor software package (SoftGenetics, State College, PA).
